# Impact of artificial intelligence on the diagnosis, treatment and prognosis of endometrial cancer

**DOI:** 10.1097/MS9.0000000000001733

**Published:** 2024-01-17

**Authors:** Samia Rauf Butt, Amna Soulat, Priyanka Mohan Lal, Hajar Fakhor, Siddharth Kumar Patel, Mashal Binte Ali, Suneel Arwani, Anmol Mohan, Koushik Majumder, Vikash Kumar, Usha Tejwaney, Sarwan Kumar

**Affiliations:** a University College of Medicine and Dentistry, Lahore; b Ziauddin Medical University; c Dow University of Health Sciences; d Karachi Medical and Dental College, Karachi, Pakistan; e Asselin Hedelin Hospital, Yvetot, France; f University of Albany, Albany; g The Brooklyn Hospital Center, Brooklyn, NY; h Medway Maritime Hospital, Kent, UK; i Chittagong Medical College, Chittagong, Bangladesh; j Valley health system, Ridgewood, NJ; k Wayne State University, Detroit, MI

**Keywords:** artificial intelligence, cervical intraepithelial neoplasia, CIN, EC, endometrial cancer

## Abstract

Endometrial cancer is one of the most prevalent tumours in females and holds an 83% survival rate within 5 years of diagnosis. Hypoestrogenism is a major risk factor for the development of endometrial carcinoma (EC) therefore two major types are derived, type 1 being oestrogen-dependent and type 2 being oestrogen independent. Surgery, chemotherapeutic drugs, and radiation therapy are only a few of the treatment options for EC. Treatment of gynaecologic malignancies greatly depends on diagnosis or prognostic prediction. Diagnostic imaging data and clinical course prediction are the two core pillars of artificial intelligence (AI) applications. One of the most popular imaging techniques for spotting preoperative endometrial cancer is MRI, although this technique can only produce qualitative data. When used to classify patients, AI improves the effectiveness of visual feature extraction. In general, AI has the potential to enhance the precision and effectiveness of endometrial cancer diagnosis and therapy. This review aims to highlight the current status of applications of AI in endometrial cancer and provide a comprehensive understanding of how recent advancements in AI have assisted clinicians in making better diagnosis and improving prognosis of endometrial cancer. Still, additional study is required to comprehend its strengths and limits fully.

## Introduction

Highlights

- Artificial intelligence (AI) applications in endometrial carcinoma include diagnosing and staging through analysis of histological slides, assisting in robotic surgery, and predicting prognosis based on tumour characteristics and lymph node metastasis.
- Challenges in implementing AI in healthcare include data security, algorithm bias, and ethical considerations such as privacy and transparency.
- The potential of AI in improving endometrial carcinoma diagnosis and treatment is significant, but further research is needed to validate its capabilities and address ethical concerns for its responsible use.

Endometrial cancer (EC) is one of the most typical cancers of female origin, and it has a survival rate of 83% after 5 years of being diagnosed and has a high prevalence in developed countries like Europe and North America^1,2^ which can be attributed to sedentary lifestyle and genetics. EC has been classified into two types depending upon their reliance on oestrogen for decades^1,2^. Type 1 EC endometrial cancer is considered oestrogen-dependent, while type 2 is oestrogen independent^3^. Both of these types can be belligerent, with a bad prognosis^1^. Other classifications include histological ones such as epithelial carcinomas, mixed and epithelial, mesenchymal tumours, endometrial stromal and smooth-muscle tumours, gestational trophoblastic diseases, and other malignant tumours^4^. The recent method of classifying EC endometrial cancer is based on genomic and molecular alterations along with their biomarkers, and it has shown a much better impact in predicting treatment prognosis in separate subclassifications of disease^2^ as shown in (Table 1).

**Table 1:** Histopathological and biochemical features of endometrial cancer

Stages		Description	Prognosis	Metastasis	Biological markers	
Stage I^5^		Restricted to the uterus and ovary	Good	Not common	ER/PR expression. PTEN expression. DNA MMR loss. Ki-67/MIB-1	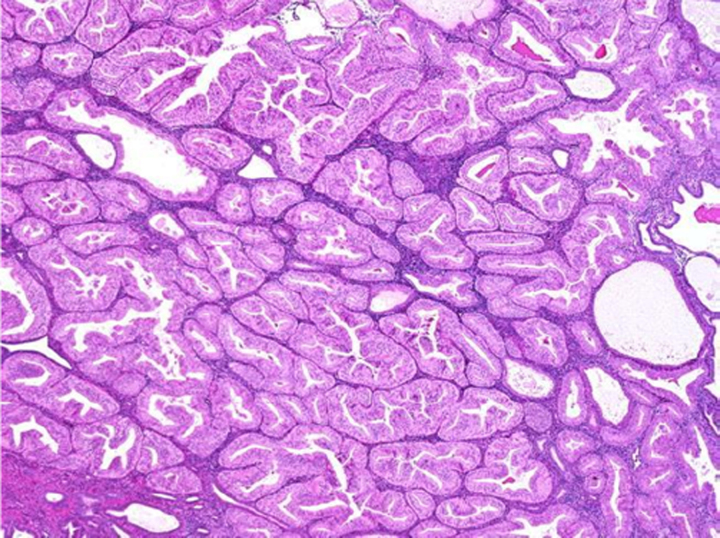
	IA	Disease confined to the endometrium or characterized by a non-aggressive histological type, such as low-grade endometrioid, exhibiting invasion of less than half of the myometrium with either no or focal lymphovascular space invasion (LVSI), indicating a favourable prognosis.				
	IA1	It characterized by non-aggressive features, is restricted to either an endometrial polyp or confined solely to the endometrium.				
	IA2	Histological types characterized by non-aggressive features, affecting less than half of the myometrium, with either an absence of LVSI or the presence of focal LVSI				
	IA3	Mild endometrioid carcinomas confined to the uterus and ovary				
	IB	Histological types characterized by non-aggressive features, involving infiltration of half or more of the myometrium, and displaying either no LVSI or limited focal LVSI				
	IC	Histological types with an aggressive nature, either restricted to a polyp or confined within the endometrium				
Stage II^5^		Infiltration into the cervical stroma either without extension beyond the uterus, or in conjunction with significant LVSI, or involving aggressive histological types accompanied by invasion into the myometrium	Variable	Not common	ER/PR expression. PTEN expression. DNA MMR loss. Ki-67/MIB-1	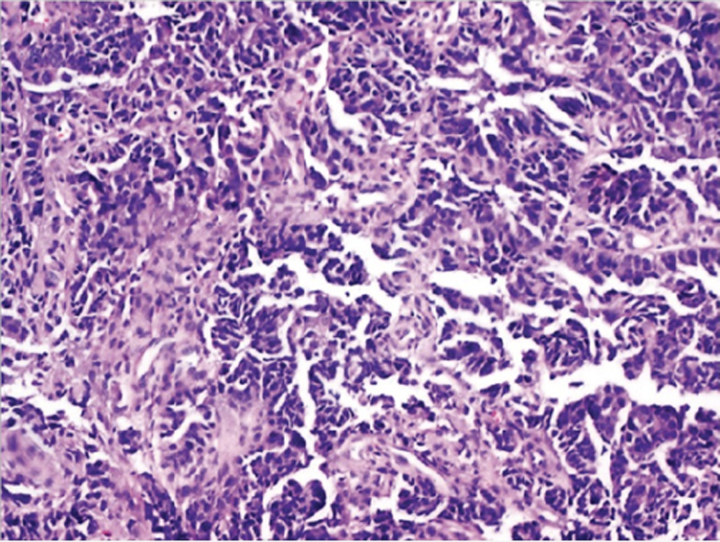
	IIA	Infiltration into the cervical stroma by histological types that are not considered aggressive			.	
	IIB	Significant LVSI in histological types that are not considered aggressive			.	
	IIC	Histological types demonstrating aggressiveness, along with any infiltration into the myometrium			.	
Stage III^5^		The dissemination of the tumour, irrespective of its histological subtype, within the local and/or regional areas.	Not good	Common	ER/PR expression. PTEN expression. DNA MMR loss. Ki-67/MIB-1	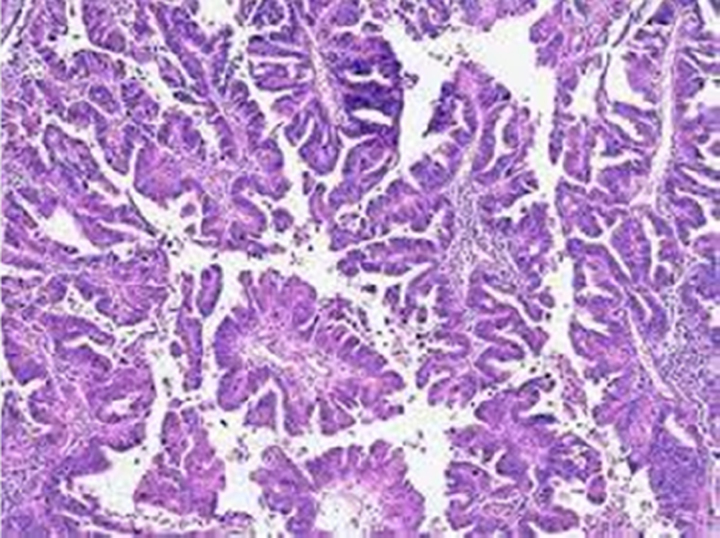
	IIIA	Infiltration of the uterine serosa, adnexa, or both through direct expansion or metastatic spread				
	IIIA1	Spread to ovary or fallopian tube				
	IIIA2	Inclusion of the uterine subserosa or dissemination within the uterine serosa				
	IIIB	Metastasis or the direct extension to the vagina, parametria, or pelvic peritoneum has occurred.				
	IIIB1	Metastasis or direct spread to the vagina and/or the parametria				
	IIIB2	Metastasis to the pelvic peritoneum				
	IIIC	Metastasis to the pelvic or para-aortic lymph nodes or both				
	IIIC1	Metastasis to the pelvic lymph nodes				
	IIIC1 i	Micrometastasis				
	IIIC1 ii	Macrometastasis				
	IIIC2	Spread to the lymph nodes near the aorta extending to the renal vessels, with or without involvement of the pelvic lymph nodes.				
	IIIC2 i	Micrometastasis				
	IIIC2 ii	Macrometastasis				
Stage IV^5^		Extension to the mucosa of the bladder and/or intestines and/or distant metastasis.	Not good	Common	ER/PR expression. PTEN expression. DNA MMR loss. Ki-67/MIB-1	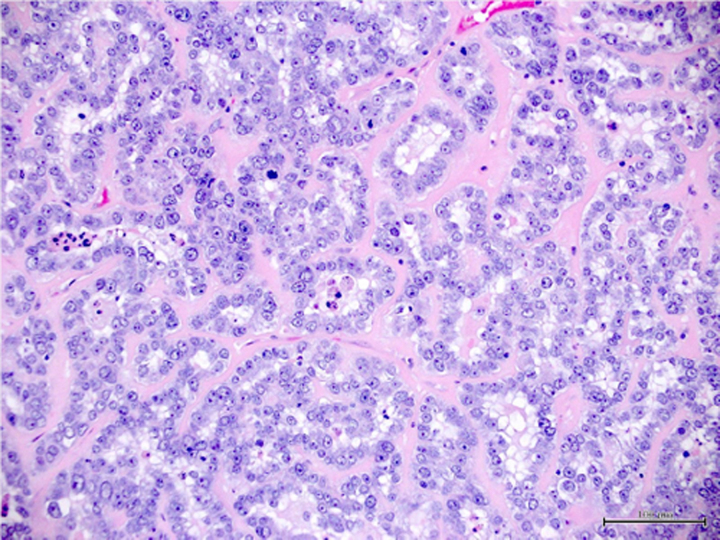
	IVA	Invasion of the bladder mucosa and/or the intestinal/bowel mucosa				
	IVB	Abdominal peritoneal metastasis beyond the pelvis				
	IVC	Distant metastasis, including metastasis to any extra-or intra-abdominal lymph nodes above the renal vessels, lungs, liver, brain, or bone				

The treatment interventions for EC endometrial cancer are various, including surgery, chemotherapy, and radiotherapy^6^. The choice of intervention was previously dependent on specific prognostic criteria, including age, FIGO staging, the extent of invasion, and metastasis into the regional and distant lymph nodes. However, major revisions are needed to achieve higher levels of diagnostic accuracy^1^. Endometrial carcinomas are difficult to diagnose because of the varying histologic criteria and differences in opinion among gynaecologic pathologists. This can result in under- or overtreatment, which can delay diagnosis and raise medical expenses^7^. Therefore, ancillary methods for the discrimination of the subtypes of cervical and endometrial cancers, and also the origin of the cancers are necessary to improve treatment decisions. Many pathologic laboratories have begun implementing digitized diagnosis procedures as a result of whole-slide images (WSIs) becoming approved for primary diagnostic uses^8^.

When it comes to application of artificial intelligence (AI) in gynaecological malignancies, it remains an untapped area for the most part. Advances in artificial intelligence have rarely been made in gynaecologic oncology. Compared as compared to other areas of healthcare. Nevertheless, precise diagnosis or prognosis prediction significantly impacts the treatment of gynaecologic cancers similar to other neoplasms. AI is mainly used for diagnostic imaging data which further aids in and predicting the clinical course^9^.

Previously AI was considered associated with a drawback that it would only work when given detailed instructions, and it will be hard to reciprocate extensive knowledge into algorithms, but now it has been discovered that AI can translate and process enormous amounts of information in a fraction of time as it has now employed a developed a deep learning (DL) method. The application of AI in the field of oncology is associated with diagnosing imaging techniques, including computed tomography (CT), MRI, ultrasound, pathological imaging, and omics data^10^. Multiomics methods can identify abnormalities in numerous biological systems, providing a more comprehensive view of the issue. However, they offer a lot of information that needs to be processed and further integrated before analysis. There are numerous multiomics dataset repositories, including ones for endometrial cancer data, as well as portals that enable the analysis and visualization of multiomics data, such as Oncomine, UALCAN, LinkedOmics, and miRDB. Research on endometrial cancer has also used multiomics techniques to find new molecular indicators and treatment targets^11^. AI can also be used to image colposcope-guided CIN cytology images due to its quality of deep learning^4^. In gynaecologic oncology, cervical cancer has been the subject of more outstanding research than ovarian and endometrial cancers. While diagnoses were primarily utilized to investigate ovarian cancer, prognoses were predominantly used to study cervical cancer. Because there was barely any research, it was unclear how well the study approaches for uterine sarcoma and endometrial cancer were performed^9^.

### Overview of AI and its applications in medical imaging

AI is a field of science and engineering, and it works in a manner that lets computers program in a way that human seems to be clever. AI helps the clinician diagnose, make therapeutic interventions, and predict the prognosis^12^. The implementation of AI in medicine has two parts: the virtual and the physical. The virtual component includes machine learning or deep learning, while the physical part includes care bots^13^. Deep learning uses artificial neural networking to mimic mathematical models as human brain neurons^10^. An ANN can learn from repetitive modifications and its experience. ML absorbs newly acquired information and either work works on a simple decision-making tree or on DL, which uses multiple data-creating patterns via neural networking. DL reprocesses a large amount of data and evaluates it numerous times before giving an outcome^14^. The computer-aided vision allows AI to perform human-driven tasks^15^. Computer vision mainly performs tasks such as comprises identifying, analyzing, and processing medical diagnostic images and videos. It works in ways similar to similarly to humans in finding the relevant results findings in medical images. Computer vision application in medicine is associated with reading radiographs, MRI, mammography, tomography, and echocardiograms^16^.

The most amount of success AI gained is in the field of radiology. The aim of AI in radiology is to minimize the workload of radiologists and eliminate the errors created by untrained radiologists; speeding up the diagnosis is quick management in emergencies. With ongoing advancements, AI can detect breast, bone, liver, lung, and cardiovascular pathologies^14^. Both the radiologists and the department appreciated the benefit of AI being able to diagnose more precisely; however, it still needs human supervision due to errors related to technicalities.AI will enable us to conduct imaging modalities like CT and MRI relatively faster^17^. As compared to human-derived imaging. In the case of U/S, which depends on the practitioner’s skill, AI-based U/S positioning and high image quality will reduce the risk of human-driven errors. Similarly, AI-driven reports also need radiographers to overlook even if they are normal^18^. Although AI is gaining popularity in cancer diagnosis and risk prediction, endometrial cancer diagnosis and risk prediction is not a field in which it has been extensively researched. Women who have limited access to quality healthcare are more likely to get endometrial cancer, which increases their mortality rate. These women are more likely to seek treatment early in the course of the disease if AI can make them aware of their risk^19^.

### The current state of endometrial carcinoma diagnosis and treatment

Artificial neural network applications in medicine have been expanding quickly. They have been used to differentiate ovarian tumours and other neoplasms prior to surgery. Despite the fact that, mathematically speaking, the distinction using neural networks would be considerably more exact than the one that could be produced by chance, the practical use of artificial neural networks in the case of endometrial cancer has been limited^20^. Every year, almost 42 000 women die from this cancer, most diagnosed after menopause^21^. Only 4% of females with EC are aged under 40 years^22^. Patients with EC typically present with a history of abnormal uterine bleeding in pre-and post-menopausal women. Generally, females present with post-menopausal bleeding, intermenstrual bleeding, prolonged menstruation duration, or a history of anovulatory cycles^23^.

The diagnosis of EC is based on histopathology and staging^24^. For assessing myometrial invasion, TVUS and MRI are essential; CT is another modality^23^. The endometrial thick of greater than 5 mm in the post and greater than 15 mm in pre-menopausal and peri-menopausal is considered to be significant measurement for investigating significant for EC^25^.

Previously D&C was performed for histological diagnosis, but now hysteroscopy-guided biopsy is considered to be the he gold standard and is suggested to be great when there is a risk of atypical endometrial hyperplasia. Hysteroscopic resection reduces the risk of spread of malignancy^26^. Other modes of getting a tissue sample for biopsy include vacuum aspiration sampling devices; France inventor Cornier E. created the Pipelle gadget in 1984. Compared to previous endometrial sample techniques, the Pipelle device, which does not require a suction pump, is more affordable, practical, and causes less patient discomfort. One of the most significant disadvantages of Pipelle is its failure to obtain adequate samples for histological examination. Some studies suggest that the provider and patient-related factors were related to sampling failure. However, German-based research indicates that the diagnosis from Pipelle and the D&C was identical in 95.5%^27^. With D&C, the chances of uterine and cervical trauma are more likely, and it is considered less cause-effective than pipeline, while pipeline has a greater failure rate^28^.

The primary imaging modalities MRI and TVUS are also associated with limitations, where TVUS is operator dependent and needs a skilled practitioner to perform. At the same time, MRI availability is still a concern. MRI is considered more specific than TVUS but can only be performed in a tertiary setup where it is available^29^. Taraboanta *et al.* ^30^ performed a retrospective cross-sectional study suggesting that outpatient endometrial sampling has low sensitivity due to failure in sampling the endometrial pathology. The specific treatment of EC depends upon the clinical stage. However, the backbone of the treatment remains the same as the traditional total abdominal hysterectomy with bilateral salpingo-oophorectomy with or without lymph node dissection. With the myometrial invasion of 50% or greater of the total width or grade 2 or 3 histology, radiotherapy is indicated to eliminate recurrence. Chemotherapy is for women with extra pelvic recurrence. Commonly used drugs for this purpose are a combination of cisplatin plus doxorubicin. Carboplatin plus paclitaxel represents an efficacious, low-toxicity regimen for managing advanced or recurrent endometrial cancer^31^. In advanced carcinomas, the adjuvant treatment is chemotherapy plus external beam radiography for the pelvis^32^. Marzi *et al*. conducted a retrospective study explaining that the hysteroscopic resection of hyperplastic areas followed by high-dosage progestin therapy is an excellent approach in patients with initial stages of EC who wish to conserve fertility^33^.

One of the major limitations with the traditional diagnosis of EC is with regards to differentiation between hyperplasia, atypia and malignancy. Endometrial hyperplasia (EH) is defined as an irregular proliferation of the endometrial glands leading to increase in the gland to stroma ratio in contrast to proliferative endometrium^34^. Five to ten percent of the causes of post-menopausal bleeding are benign endometrial hyperplasia. Malignancy must be ruled out in women with atypical endometrial hyperplasia (AEH) because up to 25% of them may also have a concurrent well-differentiated adenocarcinoma^35^. AEH is considered a pre-cancerous lesion^36^. Definitive diagnosis is made by biopsy of the endometrium on hysteroscopy^34^. Although hysteroscopy emerged as a predictive factor of endometrial cancer, its accuracy was low, raising the issue of the specific macroscopic features of endometrial cancer leading to under- or overtreatment of patients with AEH with factor risks of endometrial cancer^36^.

### AI applications in endometrial carcinoma diagnosis

Zhang and colleagues used hysteroscopic guided images of endometrial lesions to determine the presence of EC. This study included 1851 images from 454 patients, these images were first pre-processed, and a training set of 6478 images was input into a tuned VGGNet-16 model; 250 images were used to test the performance. After the model made its diagnosis, they compared it with the diagnosis given by gynaecologists. This study confirms that we can use deep learning to assess endometrial lesion images obtained via hysteroscopy^37^.

Zhao *et al.* ^38^ conducted a study in which they discovered 14 genes involved in EC, based on GEO and TCGA, and formed a diagnostic model for EC through an ANN; this model showed high sensitivity and specificity for diagnosing early EC.

MRI is one of the most used imaging methods for the detection of preoperative EC, but it is only restricted to qualitative results. Tao and colleagues use the case selection method to assess the role of deep learning on MRI assessment for diagnosing EC. AI increases the efficiency of image feature extraction and its involvement in classifying patients^39^.

AI using convolutional neural networking with MRI shows high diagnostic performance^40^. A systematic review and meta-analysis were conducted on females with uterine body cancer (carcinoma+sarcomacarcinoma sarcoma), suggesting that AI-based diagnostic assessment of radiological, histological, and biochemical modalities can improve the effectiveness of treatment and prognosis of the disease. In addition, using omics data with AI can outpass traditional classifications such as ECMO, allowing better management in every case^41^. With combined artificial intelligence and bioimpedance, we can differentiate between benign and malignant endometrial tumours, helping us decide the appropriate treatment^42^.

With the concern of screening the population for EC risk assessment, Hart and colleagues conducted a study using 7 different AI algorithms, out of which random forest was the best, and showed that AI, if used for screening, will not only be cost-effective but it will also be a non-invasive^43^ manner of screening as compared to the traditional method^44^.

Kim *et al.* ^45^ performed a retrospective study to determine the effectiveness of CT-based AI waist skeletal muscle volume; this study suggested that waist skeletal muscle can be an excellent biomarker for EC and will help predict the prognosis of the disease. Akazawa and colleagues Al tried to predict recurrence in early stage endometrial cancer using machine learning methods based on clinical data but due to the tiny quantity of the dataset, the machine learning classifiers did not perform as well as they might have. Early stage endometrial cancer recurrence might be predicted with the use of a machine learning algorithm^46^

With regards to biopsy obtained samples specifically the samples from pipelle biopsy which has higher chances of failure^27^. Pathology is increasingly digitized with slides being scanned and viewed on screens rather than through microscopes and Computerized systems based on ANNs can aid the cytological classification of endometrial nuclei and lesions with sufficient sensitivity and specificity^47^.

### AI applications in endometrial carcinoma staging, treatment and prognosis

The International Federation of Gynaecologists and Obstetricians developed a surgical system for EC in 1988, known as the FIGO staging. Before 1971 clinical staging was used for EC. In 2009 FIGO staging was revised after surveying 42 000 females who underwent the surgical staging for EC. The revised staging system further divides EC into subtypes providing a better prognosis due to effective treatment for every individual stage^48^.

Due to recent advancements, AI has many applications in the diagnosis of disease, preoperative planning, intraoperative assistance, surgical training and assessment, and robotics^49^. With use of robotics in EC treatment a significant reduction in the postoperative risk, duration of stay at hospital, lessa smaller number of lymph nodes dissected, less blood loss has been seen. Robotic surgery not only being cost effectivecost-effective but also reduces the risk of recurrence^50–52^.

EC is the most common gynaecological cancer in developed countries, with over 377 227 cases reported in 2020. The risk factor for the high occurrence of EC is associated with an increase in worldwide obesity, life expectancy, and hormone replacement therapy. The prognosis seems largely dependent on the diagnosis at a younger age, associated with an earlier disease stage, a more favourable histological subtype, and more low-grade tumours with higher surgery and lower chemotherapy rates^53^. Multiple AI-based models are used to predict the prognosis of EC but we must consider that this discipline is still developing and that we are just in its infancy. However, radionics is an up-and-coming field, and its use in endometrial cancer may alter our practices in the future^54^. Following are some studies that demonstrate the use of AI on the staging, treatment, and prognosis of EC (Table 2).

**Table 2:** Predictive models employed in staging, treatment and prognosis of endometrial cancer

Author	Country	Study design	Objective	Outcome
Lowe *et al*.^50^	United States	Non-randomized control clinical trial	To report perioperative outcomes and learning curve characteristics from a multi-institutional experience with robotic-assisted surgical staging for endometrial cancer.	Robotic technology may be an excellent intervention for EC staging, but it further requires its comparison with laparoscopy and laparotomy
Cardenas-Goicoechea *et al*.^51^	United States	Retrospective review	To compare the survival outcomes of women with endometrial cancer managed by robotic and traditional laparoscopic-assisted surgery	There were no significant differences in survival (3-year survival 93.3% and 93.6%), DFS (3-year DFS 83.3% and 88.4%), and tumour recurrence (14.8% and 12.1%) for robotic and laparoscopic groups, respectively
Göçmen *et al*.^52^	Turkey	Prospective study	To compare the results of patients on whom staging was applied by robotic-assisted laparoscopic surgery and laparotomy for endometrial cancer	The outcome of this study was in favour of robotic-assisted surgery with its advantages of less hospital stay duration, low blood loss, and less frequency of lymph node dissection
Lecointre *et al*.^54^	France	Systematic review	To investigate the contribution of radiomics on the radiological preoperative assessment of patients with EC; and to establish a simple and reproducible AI Quality Score applicable to machine learning and deep learning studies	Preliminary data indicates that these new technologies, when combined with human intelligence, can address some of the clinical problems, even though there is not enough proof to support the use of radiomics in the treatment of endometrial cancer.
Fell *et al*.^55^	Scotland	Cross sectional	To categorize endometrial biopsy whole-slide images (WSI) from digital pathology as either “malignant”, “other or benign” or “insufficient” with the help of AI	The final model accurately classifies 90% of all slides correctly and 97% of falls in the malignant class, suggesting that the use of AI in screening whole cell slides for determining their cytology is enough to implement this for the reduction of pathologists’ workload
Markis *et al*.^56^	Greece	Cross sectional	To investigate the efficacy of an artificial neural network based on multi-layer perceptron (ANN–MPL) to discriminate between benign and malignant endometrial nuclei and lesions in cytological specimens.	For the case classification based on the numeric classifier, the overall accuracy was 90.87%, the specificity 93.03%, and the sensitivity 87.79%; the indices for the percentage classifier were 95.91%, 93.44%, and 99.42%, respectively. These invented computerized systems based on ANNs can be helpful for the cytological classification of endometrial nuclei and lesions with adequate sensitivity and specificity
Bell *et al*.^57^	United States	Retrospective chart review	To compare hysterectomy and lymphadenectomy completed via robotic assistance, laparotomy, and laparoscopy for endometrial cancer staging with respect to operative and perioperative outcomes, complications, adequacy of staging, and cost	Robotic hysterectomy provides better node retrieval to laparotomy and laparoscopic procedures if performed by a skilled laparoscopic surgeon. Robotics provides the patient with a speedier recovery and reduces the risk of postoperative morbidity. Moreover, the average cost for hysterectomy and staging was highest for laparotomy, followed by robotic, and least for standard laparoscopy
Feng *et al*.^58^	China	Retrospective study	To develop a deep learning (DL) model for prediction of lymph node metastasis (LNM) based on hematoxylin and eosin (HE)-stained histopathological images of EC. The model was validated using external data	It was discovered that a novel DL-based biomarker, especially for patients in the early stages of staging, might predict metastatic status with enhanced accuracy after being trained on various histological subtypes of EC slides
Mysona *et al*.^59^	United States	Retrospective study	To determine the utility of a clinical calculator to predict the benefit of chemotherapy in stage IA uterine papillary serous cancer (UPSC)	it was discovered that a low-risk group would not benefit from chemotherapy when the relative benefits of the treatment were evaluated

AI, artificial intelligence; DFS, disease-free survival; EC, endometrial carcinoma.

## Discussion

Recent findings in AI applications in endometrial cancer indicate significant progress and potential for improving diagnostic and prognostic outcomes. A systematic review conducted by Akazawa and colleagues in 2021 analyzed the state of AI research on gynaecologic cancers, including endometrial cancer. The review encompassed studies conducted between January 2010 and December 2020. Out of 1632 articles, 71 were eligible, including 13 focused on endometrial cancer. These studies primarily utilized imaging data (49%) and value-based data (51%) as input for AI models. MRI, CT, ultrasound, cytology, and hysteroscopy data were used for imaging, while patients’ backgrounds, blood examinations, tumour markers, and pathological examination indices were used for value-based data. The main targets of prediction were definitive diagnosis and prognostic outcomes, such as overall survival and lymph node metastasis. The study also revealed certain challenges in the application of AI to endometrial cancer research. One significant challenge is the relatively small size of the datasets, with 90% of studies including less than 1000 cases and a median dataset size of 214 cases. This limitation can impact the generalizability and reliability of the AI models. Additionally, the lack of external validation datasets further hinders the robustness of the findings. The proficiency of the study design for endometrial cancer and uterine sarcoma was also unclear due to the small number of studies conducted on these cancers^9^.

Another study conducted by Erdemoglu and colleagues in 2023 focused on the prediction of endometrial intraepithelial neoplasia and endometrial cancer risks in pre-menopausal and post-menopausal women using AI methods. The research included 564 patients and employed various AI algorithms, such as random forest, logistic regression, multi-layer perceptron, Catboost, Xgboost, and Naive Bayes, for classification. The features used for modelling included age, menopause status, abnormal bleeding, obesity, hypertension, diabetes mellitus, smoking, endometrial thickness, and history of breast cancer. The results demonstrated promising outcomes, with an accuracy of 94% for predicting a pre-cancerous disease and an area under the curve (AUC) of 0.938 for the receiver operating characteristic curve. Age, BMI, and endometrial thickness were identified as significant factors associated with a high risk of developing pre-cancerous and cancerous diseases. The study indicated that AI can effectively identify women at risk of endometrial intraepithelial neoplasia and endometrial cancer, highlighting its potential for clinical decision-making and personalized risk assessment. In conclusion, recent research in AI applications for endometrial cancer shows progress in utilizing AI for diagnostic and prognostic purposes. However, challenges remain, such as the need for larger and more diverse datasets and external validation to enhance the reliability and generalizability of AI models. Despite these challenges, AI holds promise in revolutionizing the management and care of endometrial cancer patients, providing valuable insights for clinicians and improving patient outcomes^19^.

### Ethical considerations of AI

The application of AI in healthcare settings has produced more proactive approaches to patient care. This has led to the creation of new fields caused new fields, such as AI-based radionics, which uses quantitative imaging to completely quantify tumour characteristics. Machine learning-based AI poses several risks to data security. Inconsistencies in algorithms, cybersecurity flaws, unfairness, bias, and discrimination, lack of contestability, problems with legal personhood, racism, problems with human rights, problems with intellectual property, adverse effects on workers, privacy and data protection issues, liability for harm, and a lack of transparency are a few additional issues that may arise^60^.

At the ethical level, the ethical value orientation of advancing human health should be considered as the top-level design to make medical AI trustworthy. Humans continue to be the duty bearers because present medical AI does not have moral standing under the law. At the regulatory level, it is suggested to improve data quality control, eliminate algorithm bias by increasing algorithm accountability and traceability, and regulate and examine the entire AI industry’s production process. It is also vital to foster international cooperation and communication and encourage various parties to debate and assess AI’s hazards and social implications^61^. A multidimensional approach encompassing policymakers, developers, healthcare practitioners, and patients is essential to mitigate the legal and ethical issues related to AI in healthcare^62^. Privacy and surveillance, bias or discrimination, and the possible philosophic conundrum of the function of human assessment are among the legal and ethical problems that AI has brought to society^63^.

In response to the increasing worries raised by the use of AI in several industries, including healthcare, a variety of AI ethics frameworks have been released over the previous six years. The application of AI-specific ethics models in healthcare is restricted, and they are typically combined with other ethics frameworks^64^.

Privacy and surveillance, bias or discrimination, and perhaps the philosophical question of the role of human judgment are among the legal and ethical challenges that society faces as a result of AI. There is no doubt that AI has the potential to transform healthcare by generating new and important insights from the massive amounts of digital data generated during healthcare delivery. But on the one hand, the main dilemma stems from a lack of accountability regarding who will take responsibility in cases of error, security breach, and mishandling of large datasets. To fully realize AI’s promise in healthcare, the following aspects must be considered: (1) informed permission to utilize data, (2) safety and transparency, (3) algorithmic fairness and biases, and (4) data privacy^65^. AI in healthcare must adapt to a constantly changing environment with frequent disturbances while adhering to ethical guidelines to protect patients’ well-being^2^. However, an easy, key component of determining the security of any healthcare software is the ability to test the software and recognize how the software would fail. ML- Health Care Applicants, on the other hand, can be a “black box” problem, with workings that aren’t apparent to assessors, physicians, or patients. Researchers should clarify how such outputs, as well as predictions, might be included in the research. This data assists in determining the cost of the scientific trial and informs scientific research^66^.

### Challenges and future directions of AI

In recent years, AI technologies have piqued the attention of a wide range of fields, notably medicine. The expansion of medical computer hardware and software and the digitization of health-related data^67^. For more than three decades, medical imaging informatics has fuelled clinical research, translation, and practice. The advancements in related research fields that this study highlights hold the promise of revolutionizing imaging informatics as it is now practiced throughout the supply chain of healthcare by providing well-informed, more precise diagnosis, prompt prognosis, and efficient treatment planning. Medical imaging informatics makes up a sizable portion of AI-based research-driven approaches approved by the Food and Drug Administration (FDA)^68^.

Deep learning-based computer vision tasks have been built on a massive collection of real-world photos that go above ImageNet. This paradigm is unsettling because it is difficult to equal that size in the medical field. While improvements in computer efficiency and transfer learning techniques continue to be useful in medicine, we must think about how we may develop approaches that use less data to train yet still generalize effectively^68^.

Cervical cancer has been the subject of more significant research in gynaecologic oncology than ovarian and endometrial cancers. Due to the few studies completed, it was difficult to determine how well the research designs for endometrial cancer and uterine sarcoma were performed. The limitations of the studies were listed as being the short size of the dataset and the lack of a dataset for external validation^9^.

In the coming future, we can see that AI will be increasingly employed in healthcare, necessitating moral accountability. Data bias must be avoided by employing proper algorithms that are based on unbiased real-time data. Diverse and inclusive programming groups, as well as regular audits of the algorithm, including its implementation in a system, are required. While AI cannot completely replace clinical judgment, it can assist clinicians in making better decisions. If there is a lack of medical expertise in a resource-constrained setting, AI might be used to undertake screening and evaluation^69^.

## Conclusion

Certainly! AI is a valuable tool in the fight against EC. In particular, machine learning algorithms can analyze large amounts of data from patient records, and imaging studies to develop predictive models for early diagnosis and prognosis. These models can help doctors identify patients at high risk of developing EC and provide personalized treatment plans based on the patient’s characteristics and disease subtype. Additionally, AI can help doctors monitor patients’ responses to treatment and adjust their treatment plans accordingly.

AI is a useful weapon in the struggle against EC. To create prediction models for early diagnosis and prognosis, machine learning algorithms in particular can analyze vast volumes of data from patient records, and imaging investigations. AI helps the clinician diagnose, make therapeutic interventions, and predict the prognosis, preoperative planning, intraoperative assistance, surgical training and assessment A personalized treatment plan based on the patient’s features and illness subtype may be created using these models to help clinicians identify individuals who are at a high risk of getting EC. AI can also assist doctors in monitoring how patients respond to treatment and modifying their treatment regimens as necessary along with minimizing errors and deceasing workload of professionals. Staging is a critical component of EC treatment, and AI can help improve the accuracy of this process. By analyzing patient records and imaging studies, AI can help doctors determine the stage of the disease more accurately, which can help guide treatment decisions. Along with all its benefits AI has few ethical and bias related drawbacks which needs to be addressed. Overall, AI has the potential to improve the accuracy and efficiency of EC diagnosis and treatment, but more research is needed to understand its capabilities and limitations fully.

## Ethical approval

Not applicable.

## Consent

Informed consent was not required for this review.

## Source of funding

Not applicable.

## Author contribution

All authors equally contributed to this work.

## Conflicts of interest disclosure

Not applicable.

## Research registration unique identifying number (UIN)

Not applicable.

## Guarantor

Koushik Majumder.

## Data availability statement

Not applicable.

## Provenance and peer review

Not commissioned, externally peer-reviewed.
